# Van Arsdale on Obstetrical Auscultation

**Published:** 1843-10

**Authors:** 


*Van Arsdale on Obstetrical Auscultation.*—The foregoing observations on the nature and value of the pulsations of the fœtal heart, seem to establish the following propositions:

1. These pulsations are a certain sign of the existence of pregnancy and of life in the fœtus.

2. The impressions upon, and the variations in, the circulation of the mother, have no effect on that of the child.

3. The *increase* in the number of the double pulsations is of little consequence, since it rarely happens and is usually of short duration. On the other hand, a *decrease* in the number, during parturition, and especially in a protracted labor, announces danger to the child, and indicates that the labor should be terminated by instruments, by version, or such other means as the case may demand.

4. We should not expect to discover these pulsations before the completion of the first half of pregnancy. The average number of beats is 135 a minute, and they continue to increase in intensity from the moment when first heard until parturition.

5. Cases of double pregnancy may be diagnosed from hearing the pulsations of two distinct fœtal hearts, and these pulsations usually differ in number from 8 to 15.

6. The detection of these beatings is of the first importance in cases of extra-uterine pregnancy, since, if they were not discovered, the case might be mistaken for a fibrous tumor of the ovaries or a simple cyst, and be treated as such.

7. They are discovered at a point corresponding with the dorsal præcordial region of the fœtus.

8. To discover the presentation, we have to draw an imaginary *horizontal* line, dividing the uterus into two equal parts. When the head presents, the pulsations are heard in the inferior half, and when the pelvis presents, they are heard in the superior half.

9. To discover the position, we have to draw an ideal *perpendicular* line, bisecting the preceding one. When the back of the child is turned towards the right side, the pulsations are heard in the right half; and when turned to the left, they will be heard in the left half.

Lastly, the entire absence of these pulsations should invariably deter us from performing the Cæsarean operation, or from making a division of the symphysis pubis; in a word, from doing anything which might prove injurious to the mother, in the vain hope of aiding the child, which we are now bound to believe to be no longer alive.—*New York Jour. of Med.*, September, 1843.

